# Fast and Accurate Disulfide Bridge Detection

**DOI:** 10.1016/j.mcpro.2024.100759

**Published:** 2024-04-02

**Authors:** Søren Heissel, Yi He, Andris Jankevics, Yuqi Shi, Henrik Molina, Rosa Viner, Richard A. Scheltema

**Affiliations:** 1 Proteomics Resource Center, The Rockefeller University, New York, New York, USA; 2 Thermo Fisher Scientific, San Jose, California, USA; 3 Biomolecular Mass Spectrometry and Proteomics, Bijvoet Center for Biomolecular Research and Utrecht Institute for Pharmaceutical Sciences, University of Utrecht, Utrecht, The Netherlands; 4 Structural Proteomics Group, Department of Biochemistry and Systems Biology, University of Liverpool, Liverpool, UK

**Keywords:** EThcD, FAIMS, disulfide bridge, XlinkX/PD, MAAH

## Abstract

Recombinant expression of proteins, propelled by therapeutic antibodies, has evolved into a multibillion dollar industry. Essential here is the quality control assessment of critical attributes, such as sequence fidelity, proper folding, and posttranslational modifications. Errors can lead to diminished bioactivity and, in the context of therapeutic proteins, an elevated risk for immunogenicity. Over the years, many techniques were developed and applied to validate proteins in a standardized and high-throughput fashion. One parameter has, however, so far been challenging to assess. Disulfide bridges, covalent bonds linking two cysteine residues, assist in the correct folding and stability of proteins and thus have a major influence on their efficacy. Mass spectrometry promises to be an optimal technique to uncover them in a fast and accurate fashion. In this work, we present a unique combination of sample preparation, data acquisition, and analysis facilitating the rapid and accurate assessment of disulfide bridges in purified proteins. Through microwave-assisted acid hydrolysis, the proteins are digested rapidly and artifact-free into peptides, with a substantial degree of overlap over the sequence. The nonspecific nature of this procedure, however, introduces chemical background, which is efficiently removed by integrating ion mobility preceding the mass spectrometric measurement. The nonspecific nature of the digestion step additionally necessitates new developments in data analysis, for which we extended the XlinkX node in Proteome Discoverer to efficiently process the data and ensure correctness through effective false discovery rate correction. The entire workflow can be completed within 1 h, allowing for high-throughput, high-accuracy disulfide mapping.

## Highlights

• MAAH is used for fast and nonspecific proteolysis with intact canonical disulfides.
• Background ions from MAAH are removed using FAIMS, increasing disulfide bond IDs.
• EThcD along with a novel data analysis platform allows for disulfide mapping in 1 h.

## Graphical Abstract

**Figure:**
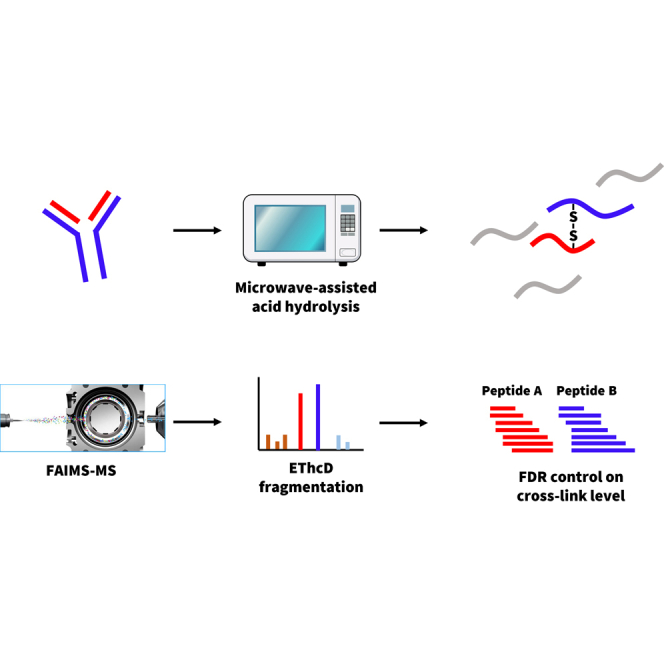

## In Brief

A method for fast and accurate disulfide mapping using MAAH and FAIMS has been developed. Using this method, we generate hundreds of overlapping peptides from each protein while keeping disulfide bonds intact. Chemical noise from the hydrolysis process is removed using FAIMS, and peptides are fragmented with EThcD. Additional developments were made to the XlinkX node in Proteome Discoverer for handling such data. The method was tested using purified proteins and allows for accurate disulfide mapping in 1 h.

Protein expression describes the intricate processes through which proteins are generated, modified, and regulated in living organisms. Within the wider context of protein research and pharmaceutical applications, these processes are utilized by researchers to direct organisms to produce desired proteins in a process called recombinant expression. Here, plasmids are introduced and encoded with the desired protein sequence that, after introduction, is automatically translated into proteins in large amounts. Different organisms can be selected for protein production, each with their own benefits. For example, *Escherichia coli* are the most commonly used organism as it rapidly expands and represents the most cost-effective option. This organism, however, lacks the capabilities to introduce complex posttranslational modifications (PTMs) like glycosylation, which can be essential for bioactivity. Other organisms, like Chinese hamster ovary cells or insect cells, are used in those cases, although these exhibit slower rates of expansion resulting in a higher cost. The advent of recombinant expression has been instrumental to kick-start the biopharmaceutical revolution, which has led to a rapid expansion that is still ongoing. Notably, between the years 2018 and 2022 alone, a total of 180 novel protein products secured regulatory approval (1). Analysts anticipate further growth due to the mounting incidence of cancer, hereditary disorders, and autoimmune maladies, complemented by the approval of numerous therapeutic interventions that modify the course of these afflictions.

An essential step in protein production is quality control of the product. During this step, the protein is investigated for multiple critical quality attributes to ensure bioactivity and, in the case of biopharmaceuticals, that they cause no immunological issues. Sequence fidelity tests determine whether the proteins have the intended amino acid sequence. Problems that can occur include point mutations or, in the case of eukaryotic cells, splice variants, which can cause loss of activity. Typically, mass spectrometry (MS) approaches are used for this step (2). Structural assays determine whether the secondary and tertiary structure of the protein are correct, which is often essential for bioactivity. Many structural biology techniques are employed here (3), the most common being NMR (4, 5), while size-exclusion chromatography provides information on the molecular weight and presence of protein aggregates (6). Finally, the presence and correct location of the required PTMs is verified. Typically MS approaches are used.

Disulfide bridges are covalent links between two cysteine residues existing commonly within the same polypeptide chain (intrachain links) and less frequently between two polypeptide chains (interchain links). This PTM is facilitated in the endoplasmic reticulum by the enzyme protein disulfide isomerase and is among the most common PTMs (7). Present mostly in secreted proteins (a large source of biopharmaceuticals), disulfide bridges are critical to the protein, and incorrect disulfide structures may lead to degradation, loss of function, or diseases (8). Disulfide mapping is one of many characteristics considered as quality attributes that must be thoroughly characterized during protein production. Mismatch of the disulfide bridges (disulfide scrambling) can cause a complex impurity profile and decreased efficacy of the biopharmaceutical which is why official guidelines call for disulfide bridge analysis during production. It is, however, not trivial to map the often-complex disulfide networks, making disulfide mapping important in both academic and industrial settings.

Several techniques within structural biology have been applied to detect disulfide bridges, such as X-ray crystallography (9) and NMR, however these are costly to execute both in terms of material needed and time spent on the analysis. In recent years, LC-MS has emerged as a powerful additional technique for studying disulfide dynamics. In the classic bottom-up proteomics workflow, proteins are reduced and alkylated, during which, disulfide bonds are disrupted and permanently blocked from reforming and finally digested into peptides with proteases such as trypsin. The reduction and alkylation serve to denature the protein and increase the accessibility for the protease. After desalting, the peptide mixture is separated by liquid chromatographyand finally analyzed by MS, where the peptides are fragmented along the peptide backbone in the gas phase (10). Disulfide bridges can be detected with LC-MS by either employing isotopically labeled alkylation agents to reduced and nonreduced protein samples to elucidate which cysteines are engaged in disulfide bonds and quantify the levels at which each residue exists as a free thiol (11), although this does not provide details on the specific disulfide pairs. Proteins may also be subjected to a step-wise reduction using an initial weaker concentration of reducing agent, followed by alkylation, reducing, and alkylating only a subset of the disulfide bonds. This is followed by a complete reduction and alkylation employing a different alkylation agent (12, 13). Disulfide bonds may also be elucidated by direct detection, where bonded peptides are identified, allowing for mapping of disulfide pairs. This strategy requires processing the proteins under nonreducing conditions (14), which may influence digestion efficiency unless a chaotropic agent is added for denaturation. Alkylation of free thiols may still be performed to identify nonbonded cysteines. Alternatively, disulfide bonds may be reduced on-the-fly by UV-photo dissociation (15, 16) and immediately alkylated *via* a postcolumn microreaction cell (17) for disulfide mapping or chemically reduced postcolumn (18).

The unambiguous determination of disulfide bridges through MS remains challenging due to several factors such as poor fragmentation properties of linked peptides. Additionally, a limited set of data analysis tools are currently available capable of dealing with the complexity of the data (unspecific digestion and alternative fragmentation techniques) while delivering low false positive rates. Researchers commonly digest the protein(s) using trypsin, but the alkaline pH optimum makes the disulfide bridges prone to scrambling, leading to incorrect results (19). The aspartic protease pepsin has a lower cleavage specificity than that of trypsin (20), and, with a pH optimum at 1 to 2 (21), pepsin remains active at conditions, where disulfide scrambling is less likely to occur. Pepsin has therefore been used as a more favorable alternative to conventional tryptic digestion in disulfide mapping (22, 23). Proteins however can also be cleaved nonenzymatically by strong acid under high temperatures either to their individual amino acid components (24) or to peptides with optimal properties for mass spectrometric detection (25), a process that may be accelerated by applying microwave energy (microwave-assisted acid hydrolysis [MAAH] (26, 27, 28)). MAAH carried out with TFA allows for hydrolysis in less than 10 min and is able to provide extensive sequence coverage (27). As the cleavage specificity is practically random, peptide generation is not reliant on the protein sequence as is the case with conventional proteolytic digestion and a high degree in overlap over the full sequence is typically achieved. MAAH has furthermore shown potential for disulfide mapping (29). However, the nonspecific cleavage pattern combined with combinatoric analyses make data analysis challenging.

In this work, we inspect the digestion properties of MAAH on a simple protein system, lysozyme C and find that we obtain suitably large, interpretable peptides and high sequence coverage while retaining the disulfide bridges. By interpreting the fragmentation spectra recorded of disulfide-bridged peptide pairs, we show that electron transfer higher energy dissociation (EThcD) produces highly informative fragmentation spectra of disulfide-bridged peptides resulting from our nontryptic approach. Next, we inspect the results from a therapeutically relevant protein—Trastuzumab—and find that the digestion approach results in a large amount of background noise hiding many of the disulfide-bridged peptide pairs. By integrating field asymmetric ion mobility spectrometry (FAIMS), which has previously shown potential in enhancing data quality in cross-linking mass spectrometry studies (30), we efficiently remove this background bringing all signals into view. The unspecific nature of the digestion necessitated optimizations to our data analysis software XlinkX (https://www.hecklab.com/software/xlinkx/) (31) both in the search as well as the false discovery rate (FDR) control, for which all data presented in this study was used to develop an approach, integrating an open search option. Next, we present data where we show our approach successfully detects disulfide scrambling in an experiment, where peptides from lysozyme C were induced to undergo scrambling. Finally, we show that our approach successfully detects and quantifies all disulfide bridges on a very short-time scale in therapeutically and structurally relevant proteins, trastuzumab and integrin α-IIb.

## Experimental Procedures

### Experimental Design and Statistical Rationale

The material used consists of single batches of purified proteins and therefore no biological replicates were used. All samples were injected multiple times to ensure consistency. All results presented in this study originate from representative single replicates. In cases where various conditions are tested against controls, the controls are obtained without the parameter being tested. For the scrambling experiment, the high-pH, high-temperature samples were compared to a hydrolyzed control sample that had been kept at acidic pH from hydrolysis to injection onto the mass spectrometer. For evaluation of FAIMS, the no-FAIMS sample was analyzed with the same chromatographic and mass spectrometer acquisition settings but omitting FAIMS.

### Chemicals and Reagents

Chicken lysozyme C (L-7651) was purchased from Sigma-Aldrich. Trastuzumab was generously donated by a pharmaceutical company and human integrin α-IIb was purified from human platelets. TFA, LC-MS grade was purchased from Thermo Fisher Scientific.

### Microwave-Assisted Acid Hydrolysis

Twenty micrograms of dry protein sample was dissolved in 40 μl 25% TFA in a 1.5 ml low-binding polypropylene vial (Eppendorf). The vial was sealed with a micro tube cap lock (Scientific Specialties) and additionally secured with tape before placing it in a bubble rack, which was placed in a 1000 ml beaker containing 200 ml demineralized water. The beaker was positioned off-center in a household microwave oven (General Electrics, model PEM31DMWW), and hydrolysis was performed using the standard setting at 800 W for 15 min (except for when hydrolysis time was evaluated, where 5, 7.5, 10, and 15 min were selected). Hydrolyzed protein was quickly dried by vacuum centrifugation and dissolved in LC-MS loading solvent (0.1% TFA) prior to injection on the mass spectrometer.

### Assessing Disulfide Scrambling

Chicken lysozyme C was acid hydrolyzed and purified by in-house constructed zip-tips. Aliquots were dissolved in 50 mM triethylammonium bicarbonate pH 8.5 and incubated at room temperature, 37 °C, and 50 °C. Time points were taken after 1 h, 3 h, and 6 h and immediately acidified. All time point aliquots were compared to a control sample, which had kept at low-pH conditions.

### LC-MS Data Acquisition

Samples were separated by reverse-phase HPLC using a Thermo Fisher Scientific Vanquish Neo system connected to an EASY-Spray PepMap RSLC C18 column (0.075 mm × 250 mm, 2 μm particle size, 100 Å pore size (Thermo Fisher Scientific)) at 250 nl/min flow rate. The hydrolyzed samples were analyzed on the Orbitrap Eclipse Tribrid mass spectrometer coupled with FAIMS Pro Duo interface. Reverse-phase separation was accomplished using a 20, 30, or 60 min separation gradient (plus 10–20 min equilibration phase) of 4 to 40% solvent B (A: 0.1% formic acid; B: 80% acetonitrile, 0.1% formic acid). FAIMS was set at standard resolution with 3.9 L/min total carrier gas flow and with 2 CV (−50/−60 or −60/−75) method. Samples were analyzed using an EThcD-MS2 acquisition strategy with 20% supplemental activation collision energy. MS1 and MS2 scans were acquired in the Orbitrap with a respective mass resolution of 120,000 and 60,000. MS1 scan range was set to *m/z* 375 to 1400, standard automatic gain control target, 246 ms maximum injection time, and 60 s dynamic exclusion. MS2 scans in data-dependent acquisition mode (top speed 1.5 s/cv) were set to an automatic gain control target of 2e5, 118 ms max injection time, and isolation window 1.6 *m/z*. Only precursors at charged states +3 to +8 were subjected to MS2. LC-MS acquisition settings for the hydrolysis-time evaluation and evaluation of modification landscape can be found in Supplementary Methods.

### Implementation of an Open Search Engine

We implemented an open search engine modeled after the approach described for MSFragger (32). To speed up fragmentation spectrum annotation, we also make use of a fragment index (Supplemental Fig. S1*A*), where each theoretical fragment is maintained in a sorted list that is binned on 0.01 Da intervals. This setup allows for almost instantaneous extraction of peptides matching a particular fragment. All fragments in a spectrum can then be annotated with peptide identities and a list constructed on the most likely peptide identities. To tie everything together, additional meta-data about the protein sequences, modifications, and the total peptide mass (Supplemental Fig. S1*B*) are required. For the initial identification, we utilize the hyper-score calculation integrated in Sequest (33). Based on this score, we assemble a top ten list of the best peptide identities and rescore this with scoring routine adapted from Olsen and Mann (34) to obtain the final peptide identity.

To investigate the performance of the engine, we compared the results from a HeLa cell tryptic digest to those obtained with Mascot or Sequest, frequently used peptide search engines in Proteome Discoverer. We identified approximately 75% of the spectra identified by Mascot along with a very low percentage of additional identifications uniquely identified by our search engine (Supplemental Fig. S1*C*). The remaining fraction of Mascot-identified fragmentation spectra were also correctly identified by our engine but discarded during the FDR control step due to low quality. We conclude therefore that our linear search engine works properly.

The integration into the crosslink search workflow is described in detail in the Results section Search Engine Optimization.

### Data Analysis

**Fig. 1:**
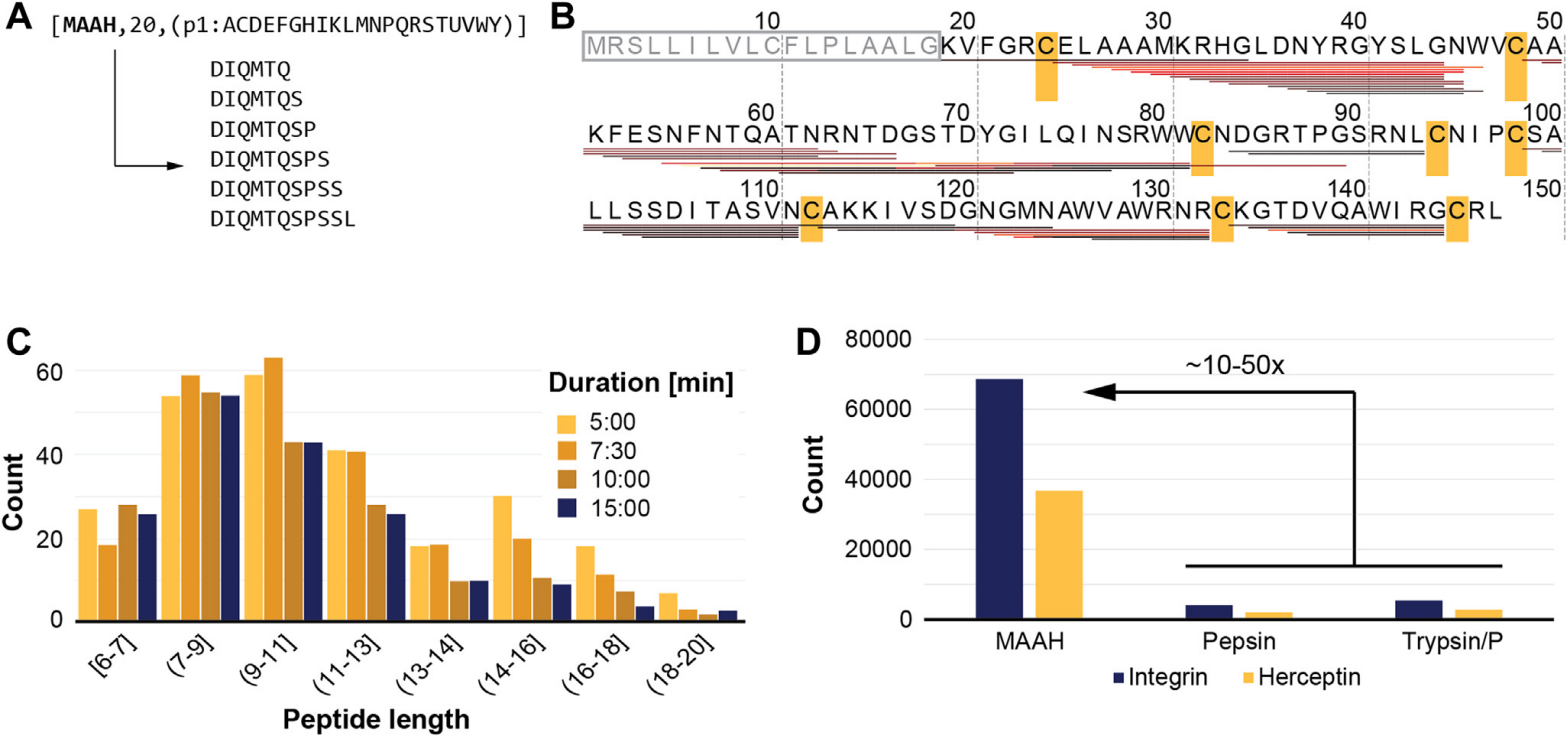
**MAAH digestion properties.***A*, cleavage settings denoted as [NAME= MAAH], [MISSED CLEAVAGES= 20], and [SPECIFICITY= all amino acids] in ExPASy PeptideCutter notation. Below are the examples of theoretical peptides. *B*, sequence coverage obtained with peptides of lysozyme C grouped on first position only. *Heat colors* denote the number of peptide-spectrum-matches at each peptide (ranging from *black* (n = 1) → *red* (n = 100) → *yellow* (n = 250)). Positions of Cys residues involved in canonical disulfide bridges highlighted in *yellow*. Signal peptide in *gray letters* inside a *gray box*. *C*, peptide lengths for lysozyme C for different hydrolysis times. *D*, effect of protease specificity on the number of theoretical peaks (or search space) generated by the XlinkX/PD search engine. MAAH, microwave-assisted acid hydrolysis; XlinkX/PD, XlinkX node embedded in Proteome Discoverer.

**Fig. 2:**
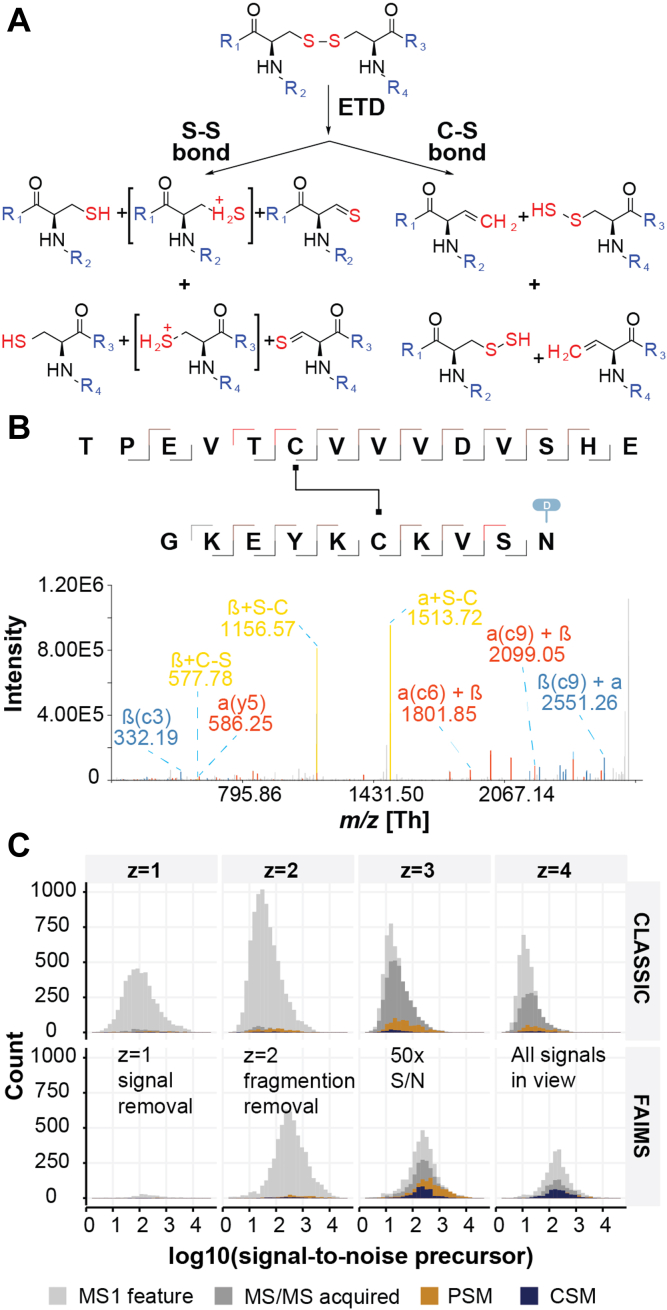
**Mass spectrometry optimizations to support disulfide bridge detection.***A*, ETD-driven gas-phase disulfide bridge reduction. *B*, EThcD MS/MS spectrum of disulfide-bridged peptide TPEVT*C*VVVDVSHE-GKEYK*C*KVSN originating from acid hydrolysis of trastuzumab with diagnostic peaks (*yellow*) and backbone fragmentation (*blue* and *red*). In the annotations, α denotes peptide A and β peptide B. *C*, effect of FAIMS integration into the acquisition investigated for trastuzumab as a function of signal-to-noise on signal-to-noise ratios of precursor ions and numbers of MS/MS spectra, peptide-spectrum-matches, and CSMs. CSM, crosslink spectrum match; ETD, electron transfer dissociation; EThcD, electron transfer higher energy dissociation; FAIMS, field asymmetric ion mobility spectrometry.

All data analysis, unless otherwise stated, was performed in Proteome Discoverer v. 3.1 SP1. Shortly, the processing workflow consists of the nodes (1) “Spectrum files” to connect the workflow to the raw files. (2) “Spectrum selector” to extract the fragmentation spectra with accurate precursor *m/z* and charge states. Standard settings were applied. (3) Linear peptide search engine was performed with the Mascot node. As FASTA file, we used one containing the protein under investigation, which is *in silico* digested as defined in Figure 1*A* with a minimal peptide length of six and up to 20 missed cleavages allowed. No fixed modification was set. Methionine oxidation, protein N-term acetylation, and asparagine and glutamine deamidation were set as dynamic modifications. (4) “Target decoy peptide-spectrum-match validator” was used to control for false positives at 1%. (5) “Spectrum confidence filter” was then used to remove all spectra that were confidently identified with the linear peptide search setting “Worse Than High.” (6) Finally, the XlinkX node in Proteome Discoverer (XlinkX/PD) nodes “XlinkX/PD detect,” “XlinkX/PD search,” and “XlinkX/PD validate” were connected. To define the disulfide bridge, we set up a cleavable crosslinker as H(-2). The electron transfer dissociation (ETD) cleavage products (or diagnostic ions) are defined as follows: S-S symmetrical as PeptideA=”H(1)” and PeptideB=”H(1),” S-S asymmetrical PeptideA=”H(2)” and PeptideB=,”” S-C asymmetrical full PeptideA=”” and PeptideB=”H(1) S(1)” (Fig. 2*A*). The crosslinker fragment setup in Proteome Discoverer is presented in Supplemental Fig. S5. As “Acquisition strategy” open search was used. The same protein database and fixed and variable modifications were used as defined in the linear peptide search.

For identified disulfide bridges, the software automatically calculates the occupancy rates in the crosslink table by collecting the intensities of linear peptides overlapping with the Cys positions involved in the bridge. The summed intensity of the disulfide bridge is then divided by the sum of the summed disulfide bridge intensity summed again to the summed linear peptides, making the occupancy a percentage.

All protein structures were visualized with ChimeraX (35) and the disulfide bridges mapped with the plugin XMAS (36).

## Results and Discussion

### MAAH Digestion Properties

As previously reported, MAAH with TFA has the capability to break down proteins into peptides suitable for sequencing in under 2 min (37). The drawback is that cleavage is indiscriminate along the entire protein backbone, unlike acetic or formic acid. To enable fragmentation spectrum searches, the cleavage specificity needs to be set as unspecific or in cases where the search engine does not support this, cleavage after every amino acid (as illustrated in Fig. 1*A*). To control the maximum length of a peptide, the number of allowed missed cleavages is restricted to 20 for the latter option to achieve the desired peptide lengths of 6 to 20 amino acids. By performing a linear peptide search with these settings using lysozyme C samples hydrolyzed for 10 min, we achieved 98% sequence coverage (excluding the signal peptide for which no peptides were detected) with peptide ladders across the entire protein backbone. Many of the locations are covered by multiple peptides supported by 100's of peptide spectrum matches multiplying confidence in the identifications. Linear peptide coverage is generally not observed around the cysteine residues involved in canonical disulfide bridges, suggesting that the disulfide bridges remain intact (Fig. 1*B*). This is not surprising, as the conditions for MAAH are ideal to retain the disulfide bridges due to its short reaction and the acidic environment, preventing spontaneous formation of disulfide bridges between free Cysteine residues. It is furthermore noteworthy that as the number of identified peptides for each protein is several folds higher than a typical enzymatic digestion, the signal for each peptide is diluted. To counter this, we injected higher amounts of peptide digests while still producing sharp peaks.

The presence of the extensive peptide ladders indicates that MAAH indeed cleaves after every possible amino acid residue. Investigating different hydrolysis times on lysozyme C (5, 7.5, 10, and 15 min) shows that for all times tested, the generated peptides are between 6 and 20 amino acids long with an average of ∼10 amino acids (Fig. 1*C*). Interestingly, for the shorter periods a set of larger peptides around 16 amino acids remain, pointing to incomplete digestion. This starts to improve after 10 min and the longer peptides are eliminated by the 15-min mark, which we consider the ideal timeframe. Excitingly, an average of ten amino acids falls perfectly within the ideal range for proteomic workflows, which is often thought to be between 7 to 35 residues (38). Fragment spectra from peptides that are too short often lack unique assignments to specific peptide identities, and peptides that are too long result in low quality fragmentation spectra. All characteristics combined, position MAAH as a potential idealease candidate, a digestion strategy dependent not only on specific residues but also on length or size (39). However, MAAH as a digestion strategy comes at a price. MAAH introduces modifications, which were investigated using an open search. The modifications were found to consist mainly of deamidation of N and Q, with minor contributions from other modifications, such as dehydration of S and T, oxidation of M, and loss of ammonia, which was found to affect mostly N and Q (Supplemental Fig. S2). Furthermore, oxidation of free cysteines and tryptophan residues were found to occur at very low frequencies. Only deamidation was included as a variable modification in subsequent searches as it is the most common modification and inclusion of many variable modifications would cause an unwanted expansion of the search space, affecting the false discovery rate calculations. Combined with the unspecific cleavage pattern, this leads to a substantial expansion of the theoretical search space, ranging from at least 10 to 50 times its original size (Fig. 1*D*).

### Optimal Peptide Fragmentation

It is by now well-established that under ETD conditions disulfide bridges in peptides are more readily cleaved than the peptide backbone (40). Although ETD fragmentation of peptide disulfide bonds is easily achieved, ETD and electron-capture dissociation fragmentation of disulfides in intact proteins is not (41, 42). The reaction pathway of how disulfide bonds are broken during ETD fragmentation is now well described (Fig. 2*A*) (40, 43). Disulfide bonds can go through S-S or C-S cleavages and generate diagnostic peaks of the individual peptides, facilitating improved data searching (see Search Engine Optimizations). Classical cross-linking searches uncovered “cross-linked” peptides, where the linker is defined as a disulfide bridge (loss of two hydrogens). As visible in a selected fragmentation spectrum from experiments on trastuzumab (Fig. 2*B*), we indeed find very abundant diagnostic ions corresponding to the *m/z* of the reduced, linear peptides involved in the bridge. However, with ETD alone, peptide backbone fragmentation events are not favored leading to low sequence coverage for the peptides individually. Integrating a higher energy collisional dissociation event, through supplemental activation, improves this, leading to high sequence coverage with *c*-, *y*-, and *z*-ions (22).

### Ion Mobility–Assisted Data Acquisition

During the analysis of acid-hydrolyzed proteins, we noted that the chromatograms were dominated by highly abundant singly charged ions in our experiments on lysozyme C. Apart from short, singly charged peptides, these ions are thought to originate from the hydrolysis process itself as they appear in the analyses of several different proteins. The overwhelming intensity of these ions has the potential to mask signals from disulfide-linked peptides *via* ion suppression. Based on recent reports, FAIMS has shown great potential in proteomics experiments by removing singly charged background signals (44).

To observe the ability of FAIMS background removal in a relevant protein system, we selected a therapeutic antibody. A total of 1 μg hydrolyzed trastuzumab was separated across a 60-min gradient with and without FAIMS applied. Two CV combinations of −50/−60 V and −60/−75 were evaluated against a control with no FAIMS. Interestingly, there were no obvious changes to the in the LC-MS profiles (Supplemental Fig. S3*A*), however the scores for the identification increased dramatically (Supplemental Fig. S3*B*). Although the overall intensity in the obtained chromatograms was lower when applying FAIMS, the number of triggered MS2 scans were found to be comparable (15,580 without FAIMS and 15,054 with FAIMS). The number of observed features with a single positive charge was drastically reduced at both CV combinations, while features from doubly charged species remained numerous (Fig. 2*C*). Interestingly, the signal-to-noise ratio of crosslink-spectrum-matches (CSMs) was increased dramatically for both CV combinations for charge states +3, +4, and +5, which are the common charges observed for disulfide-bridged peptides, resulting in a total 5.5-fold increase of CSMs for CV combination −50/−60 V and 4.9-fold for −60/−75 V over the NO-FAIMS control, with an increased number of CSMs associated with every disulfide bridge (Supplemental Fig. S3*C*). Excitingly, this increase brings all signals into view as evident from the transformation of a log normal distribution for the NO-FAIMS experiments to a normal distribution for the FAIMS experiments.

Compared to enzymatic digestions with trypsin, MAAH routinely allows for detection of several hundred (in some cases thousands) of peptides for each protein, meaning each residue is covered by a high number of peptides. The same is true for disulfide-bridged peptides, and the high number of repeated CSMs per link was found to be efficient in controlling FDR at the cross-link level. The increased number of CSMs obtained by adding FAIMS was thereby crucial for detecting false positives and increasing the accuracy of the strategy.

### Search Engine Optimization

**Fig. 3:**
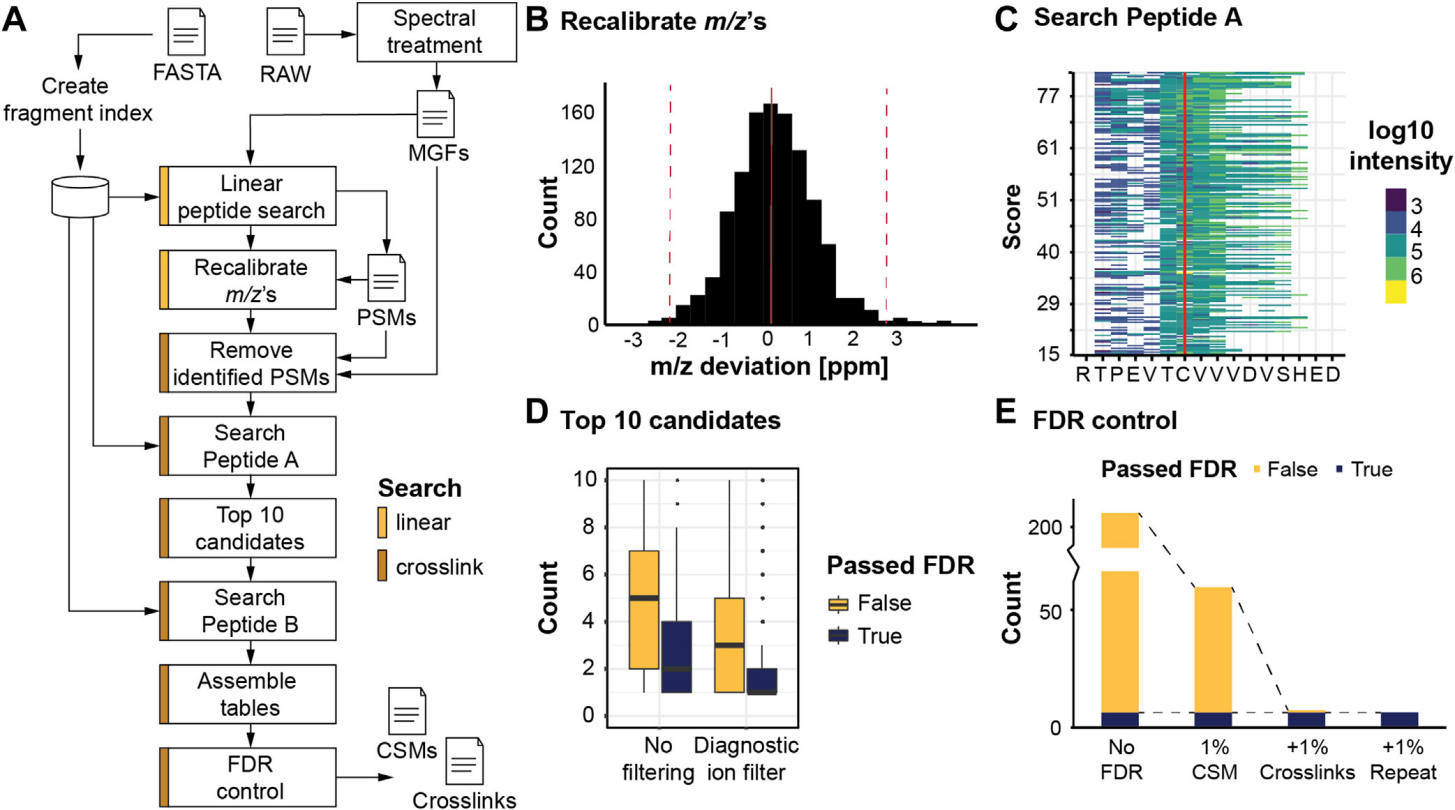
**Data processing steps and their results in XlinkX/PD.** All data were originating from acid hydrolysis of trastuzumab. *A*, workflow for processing RAW data with the challenging conditions imposed by MAAH digestion and producing the final crosslink spectrum match (CSM) and crosslink tables. Linear peptide search in *yellow*; cross-linked peptide search in *brown*. *B*, mass accuracy of precursor *m/z* values after recalibration in Proteome Discoverer. The boundaries (*dotted red line*) are estimated with interquartile range fences. *C*, sequence coverage of peptide RTPEVTCVVVDVSHED with c-ions obtained during the step “search peptide A.” Each line is a single fragmentation spectrum, 218 identifications in total. *D*, the rank of the correct peptide A after sorting on score with or without considering the diagnostic peaks in EThcD MS/MS. *E*, effectivity of the implemented FDR approach on CSM and crosslink level for trastuzumab. FDR, false discovery rate; EThcD, electron transfer higher energy dissociation; MAAH, microwave-assisted acid hydrolysis.

To increase the efficiency of data analysis and overcome the challenges posed by the unspecific nature of MAAH protein digestion, we integrated an open search module into the XlinkX/PD data analysis environment (see Experimental Procedures). Previously, all XlinkX/PD workflows utilized a closed search setup, where essentially every peptide needed to be combined with every other peptide to find the matches against the single precursor mass. Despite working exclusively with purified proteins, the expanded theoretical search space (see MAAH properties) resulted into analysis times exceeding 24 h per sample using traditional methods (31, 45). To streamline the data analysis pipeline, we utilize the structure depicted in Figure 3*A*. The pipeline starts with deisotoping and cleaning of fragmentation spectra extracted by Proteome Discoverer, which already includes accurate precursor *m/z* and charge-state information. Further steps are as follows:

1. **Cluster Peaks Removal:** Noisy peaks clustering around the real peak, likely artifacts from the Fourier transform, are eliminated;
2. **Precursor Peak Removal:** Peaks larger than the precursor mass minus 2x water are removed to avoid negative impact on the hyper-score used for open search;
3. **Immonium Ions Removal:** These ions are removed based on accurate masses to avoid negative impact on the hyper-score;
4. **Spectrum Filtering:** The spectrum is filtered to retain the TopX of 20 peaks per 100 Da, a standard method to remove noise peaks;
5. **Neutral Loss Peaks Removal:** Nonannotated neutral loss peaks are removed based on mass differences, enhancing the hyper-score accuracy.

Combined, these steps yield clean and interpretable fragmentation spectra.

The second stage focuses on identifying linear peptides from the available fragmentation spectra, as these peptides are easier to identify than cross-linked pairs. After FDR control at the peptide-spectrum-match, peptide, and protein level (not shown in Fig. 3*A*), this step provides information for calibrating precursor masses (Supplemental Fig. S4, *A* and *B*). A maximum acceptable mass deviation is estimated after calibration, utilizing interquartile range fences (red dotted lines in Fig. 3*B*). The effect of applying *m/z* calibration is dramatic, as even after stringent FDR control (see next paragraph) false positives remain when calibration is not applied while only the correct disulfide bridges remain when calibration is applied (Supplemental Fig. S4*C*). For the presented data, a development version of XlinkX/PD capable of identifying linear peptides was used. However, similar results can be obtained with regards to mass calibration and removal of confidently identified spectra by using mass recalibration option and any search engine for linear peptides available in Proteome Discoverer.

In the final stage, cross-linked (or disulfide-bridged) peptides were identified. Confidently identified linear peptide spectra are removed and an open search strategy employed to identify the possible sequence of the first peptide (PeptideA) in the remaining spectra. High sequence coverage for the correct PeptideA was achieved for all spectra supported by intense fragment ions (Fig. 3*C*). This information is then used to create a list of possible peptide identities, sorted based on the hyper-score used in the open search, resulting in the correct peptide located within the top 8 in >95% of the cases. Enforcing the presence of at least one “cross-linker” cleavage product (see Fig. 2*A* for the possible products) improves the accuracy significantly, with the correct identity in over 95% of the cases located within the top 3. The second peptide (PeptideB) is identified by calculating the mass difference between the precursor and the summed mass of PeptideA and the crosslinker, followed by a classical search approach using a scoring routine (see Experimental Procedures). Due to only considering the top 10 PeptideA identities, search time is dramatically reduced from excess of 24 h to less than 5 min per raw file.

### FDR Correction

In the final stage of the analysis, we compile the complete list of identified cross-linked peptide spectra, along with their corresponding peptide pairs, into a CSM table. However, this table contains, next to the canonical disulfide bridges, numerous false positives (depicted in Fig. 3*D* for trastuzumab) that require elimination through a curation process. To achieve this, we employ a multistage strategy as outlined by Lenz *et al* (46). Initially, we identify the peptide identities for both PeptideA and PeptideB using a database containing authentic protein sequences and another with decoy sequences (comprising fully reversed protein sequences, digested into theoretical peptides). Only the top-scoring pair is retained for analysis. If either of the peptides originates from the decoy database, the identification is flagged as decoy. To establish the appropriate score cut-off, the complete list is sorted, and the number of decoy identifications is calculated for each potential cut-off, following the methodology described by Elias *et al* (47). This step eliminates more than half of the false positives, although the remaining fraction necessitates further curation. The results from the CSM table are then condensed into a crosslinks table, grouping peptide pairs based on their positions in the protein, including PTMs and missed cleavages. Similar to the CSM table, the crosslinks table is FDR controlled based on the maximum score among all entries for each crosslink, and highest scoring CSM is reported for identified unique pair of cross-linked sites. These filtering steps effectively eliminate false positive identifications, which are common due to the nonspecific nature of the digestion and the significant expansion of the search space (up to 50–100 times). Consequently, only canonical disulfide bridges remain, with just one false positive identification.

### Disulfide Bridge Scrambling during MAAH

**Fig. 4:**
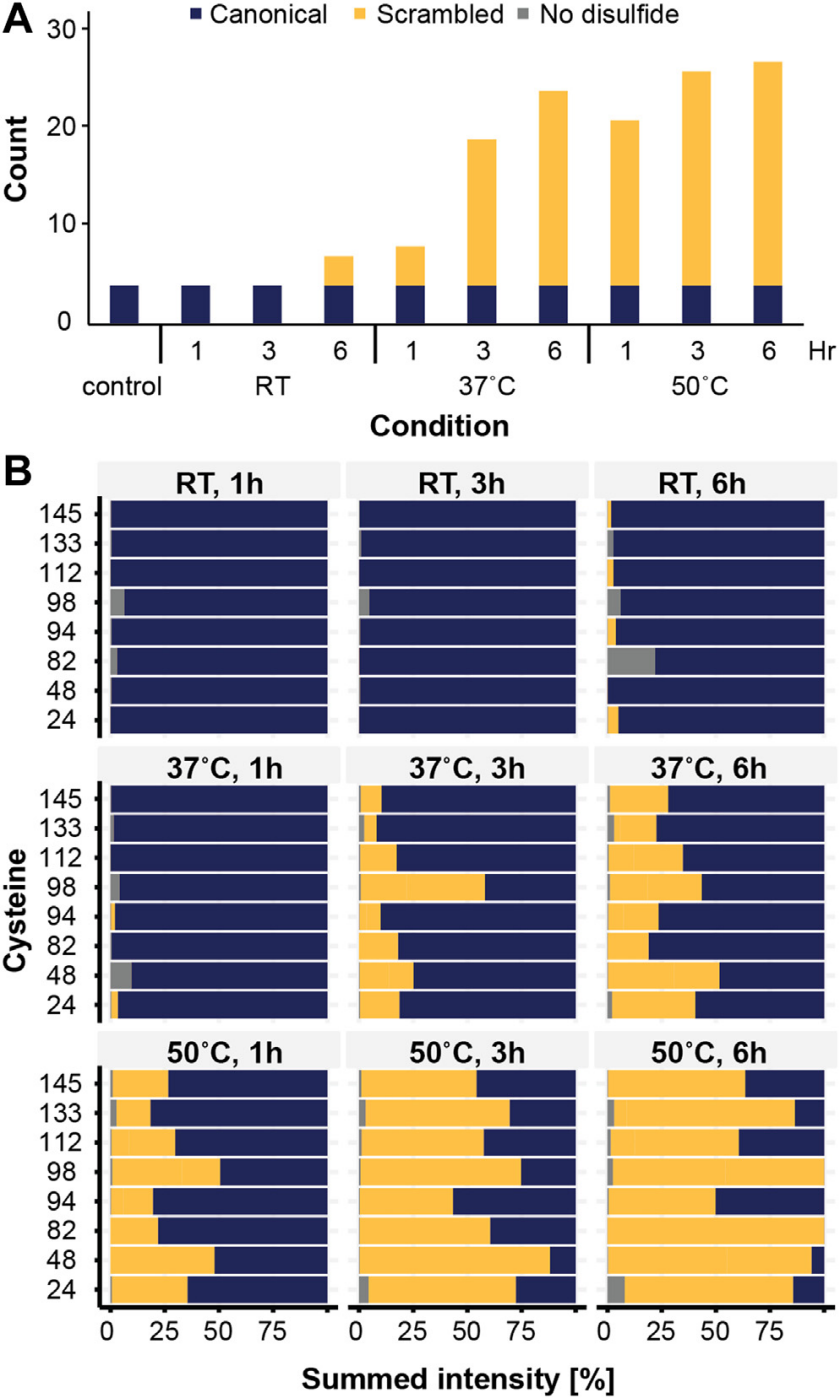
**Detection of disulfide bridge scrambling in MAAH digested chicken lysozyme C.***A*, detected disulfide bridges as a function of the used conditions (*top time, bottom temperature*). *B*, summed intensities of the detected disulfide bridges and linear peptides. MAAH, microwave-assisted acid hydrolysis.

Next, we evaluated the identified disulfide-bonded peptides to uncover the degree at which scrambling of disulfide bridges (*i.e.*, nonbiologically relevant disulfide bridges induced by incorrect folding or sample preparation issues) occurs. This attribute is critical if the workflow is to be used as a QC tool for biopharmaceutical proteins. To ascertain this, we evaluated the degree of disulfide scrambling in an acid hydrolysate of lysozyme C, which was subsequently incubated at pH 8.5 at room temperature (25 °C), 37 °C, and 50 °C for 1, 3, and 6 h; optimal conditions for free cysteines to react with one another (Fig. 4*A*). These samples were compared to a control acid hydrolysate, which had not been incubated at higher pH. As expected, we exclusively identified the four correct bridges in the control sample (48). At room temperature, however, we already observed three scrambled bridges after 6 h of incubation. At 37 °C and 50 °C, after just 1 h incubation, we observed four and 17 scrambled bridges, respectively. The number of scrambled disulfide bridges rapidly increased with longer incubation times and after 3 h at 37 °C a total of 20 scrambled disulfide bridges were identified, which is comparable to the number identified after just 1 h at 50 °C. We noted that that the intensities of the canonical disulfide bridges significantly dropped after incubation at pH 8.5. Peptides representing noncanonical links increased rapidly in intensity at 37 °C and at 50 °C. These links had reached intensities comparable to those of the canonical links within just 1 h (Fig. 4*B*).

### Disulfide Mapping of Protein Standards

To further highlight the capability of the presented approach, we characterized the disulfide bridges of proteins with a clear clinical relevance and varying complexity in their disulfide landscapes. The first, trastuzumab, is a mAb that is used to treat breast cancer for patients that are shown to be *human epidermal growth factor receptor 2* positive. The second, human integrin alpha-IIb, is expressed by a wide variety of cell types, including T cells (the NKT cells), NK cells, fibroblasts, and platelets. Integrins are involved in cell adhesion, participate in cell-surface–mediated signaling and play a critical role in platelet aggregation.

**Fig. 5:**
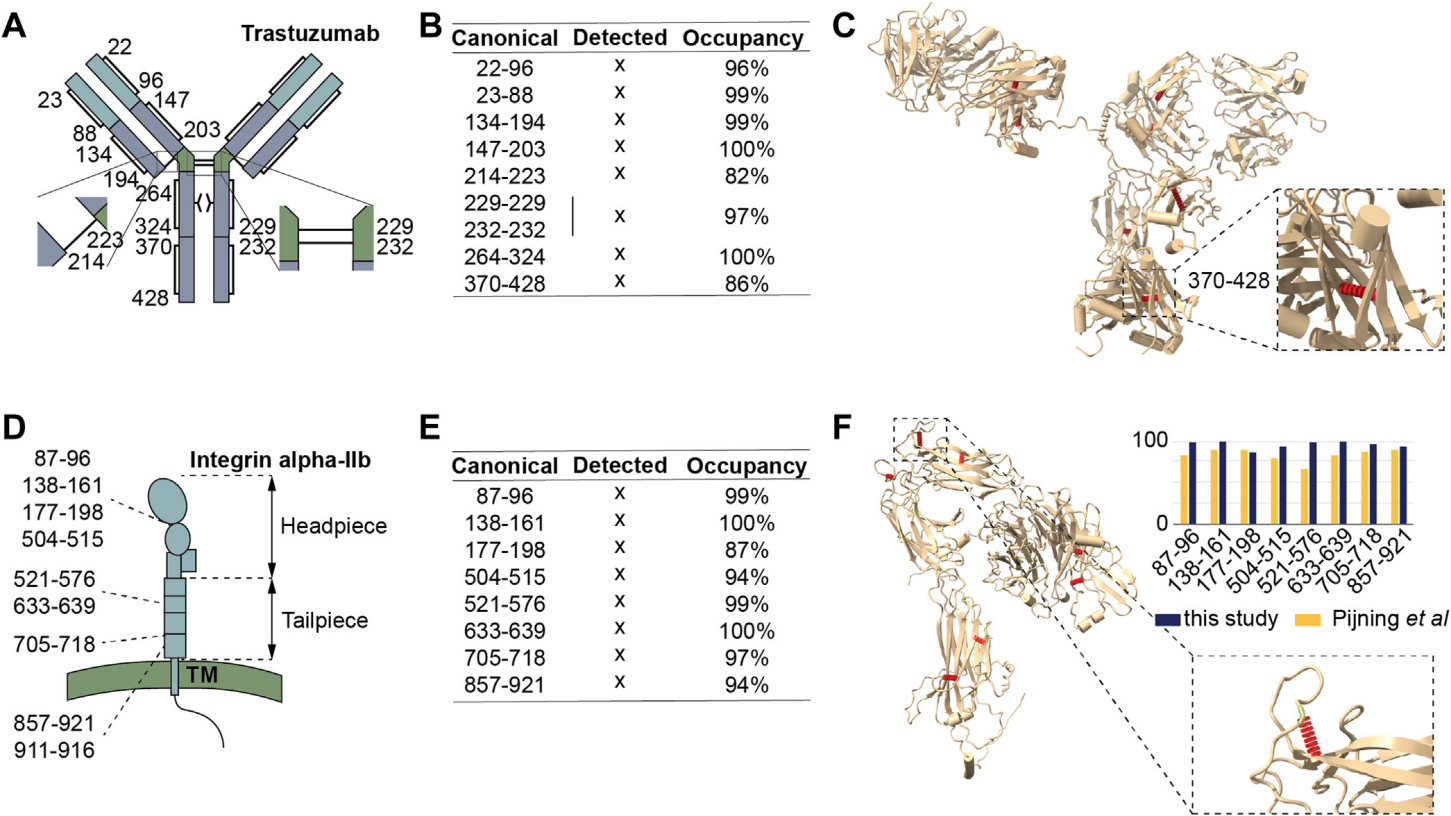
**Disulfide mapping of relevant proteins.***A*, schematic of trastuzumab with the canonical disulfide bridges indicated. *B*, all canonical disulfide bridges were detected (numbering in UniProt, for EU numbering subtract 3) and found to have near 100% occupancy rates, except for the disulfide bridges 214-223 and 370-428 (*middle panel*). *C*, structure of trastuzumab with the disulfide bridges highlighted (inset: zoom-in on the structure around 370–428). *D*, schematic of integrin alpha-IIb with the disulfides indicated in the domains. *E*, all canonical disulfide bridges were detected and found to have high occupancy rates. *F*, structure of integrin alpha-IIb with the disulfide bridges highlighted. *Top right*, comparison of the occupancy rates of this study and those obtained by Pijning *et al* (52). *Bottom right*, zoom-in on structure around Cys504-Cys515, which showed a low occupancy rate).

For trastuzumab, a total of nine disulfide bridges are described (49) which are indicated in schematic form in Figure 5*A*. From our analysis, we identify seven bridges with standard settings. The disulfide bridges at Cys229 and Cys232 are missing, which can be explained by their close spacing, resulting in peptide pairs connected by two disulfide bridges. To account for this, we added a disulfide bridge (*i.e.*, H(-2)) as variable modification, resulting in the positive identification of these disulfide bridges in a single peptide pair. As the disulfide bridges at position 229-229 and 232-232 are identified from a single peptide pair, we cannot calculate an occupancy rate for each bridge individually, but the combination has an occupancy of 97% making it likely that both are present in the vast majority of the cases (Fig. 5*B*). The remaining of the calculated occupancy rates show high occupancy for all disulfide bridges except for one. The bridge Cys370-Cys428 is in 86% of the cases occupied. It was previously shown that though removal of this disulfide bridge affects stability of the antibody, it does not structurally change the antibody to the level that intact immunoglobulin G can still be formed and the antibody remains active (50). This is well supported by its role in stabilizing two already connected beta sheets (Fig. 5*C*). Hexamerization can be affected potentially modulating complement activation, although previously it was observed that disconnected disulfide bridges can spontaneously reform in the presence of plasma (51).

For integrin alpha-IIb, nine disulfide bridges are also described that are indicated in schematic form in Figure 5*D*. With standard settings, eight were successfully detected, all at high occupancy rates (Fig. 5*E*). The disulfide bridge Cys911-Cys916 was not found in our analysis, for which previous reports also do not provide evidence of existence (52). A lower occupancy is detected for Cys504-Cys515, which maintains a small loop (Fig. 5*F*). To verify whether our observation is correct, we correlated our occupancy rates to those previously reported (Pijning *et al*, 2022, Fig. 4*A*, (52)). This study reports Cys521-Cys576 with the lowest occupancy, while the rest of the disulfide bridges correlate very well on occupancy rates.

### Analysis Time Reduction

Sample throughput is major concern in many proteomics laboratories, both industrial and academic. Given the significant speed increase in data analysis and the extreme repetition rates of identifications, we reasoned that the data acquisition time can be reduced as well. To this end, we evaluated the capabilities on lysozyme C of this approach using a 20-min and a 30-min gradient rather than 60 min, which would allow for disulfide mapping on a much shorter time scale. Considering the drastic increase in data quality attributed to FAIMS, we hypothesized that a shorter gradient would still provide extensive linkage information. Using lysozyme C we were able to cover all four disulfide bridges using the 20-min, 30-min, and 60-min gradient with 79, 135, and 217 CSMs, respectively. These results demonstrate that we can go from intact protein to complete disulfide mapping results within a single hour.

## Conclusions and Discussion

In this paper, we present an efficient and precise sample preparation, data acquisition, and analysis approach for the accurate detection of disulfide bridges. In earlier studies, researchers attempted to identify disulfide bridges through indirect means like differential alkylation or direct detection as “cross-linked” peptides (53, 54, 55, 56, 57, 58, 59, 60). Typically, these methods involved analyzing samples under unfavorable conditions. For instance, they employed traditional enzymatic reactions, where digestion occurred in a slightly alkaline pH setting. While this approach allowed the utilization of existing tools tailored for proteomics workflows, it had a drawback: it left proteins susceptible to rearrangement of their disulfide bridges, forming nonbiologically relevant connections between cysteines. To overcome this, several studies report reduced scrambling when performing digestions at pH 5 to 6, by applying proteases with a lower pH optimum such as pepsin (22, 23) or by the addition of an oxidizing agent (61). Most enzymes used in proteomics experiments however require higher temperatures and longer incubation times to ensure complete digestion and, so far, these did not gain traction for the detection of disulfide bridges. In contrast, the MAAH that is central to our approach efficiently hydrolyzes proteins in highly acidic conditions, preventing such scrambling. MAAH however produces a high abundant background, which must be removed to keep required high dynamic range for disulfide identification. Adding FAIMS as a gas filtering device improves the number of identified CSMs 5-fold (Fig. 2) and enables identification of all S-S links in the systems we investigated. EThcD has previously shown great potential for disulfide analysis (22) and as this fragmentation strategy produces not only backbone fragments but also intense ions from the intact linear peptides, the spectra were rich in information.

The required search engine optimizations were implemented in the XlinkX node in PD v. 3.1 sp1. This data analysis pipeline has for the last 7 years gone through continuous development cycles and will continue to do so. For the near future, we are extending its functionality to encompass a broader set of options. One of these will be the extension of XlinkX/PD for the extraction of higher order (more than two) cross-linked peptides. Currently, XlinkX/PD is restricted to two peptides (or dipeptide) crosslinked together. For disulfide-bridged peptides, more peptides need to be supported. A famous example is insulin, where the disulfide bridges intertwine around closely spaced positions. For the current implementation, a workaround enables their detection.

Our comprehensive protocol not only accelerates the sample preparation, data acquisition, and data analysis but also guarantees precise and dependable identification of cross-linked peptides in intricate samples. This makes it an invaluable tool for high-throughput quality control applications, which is an increasingly important part of the biopharmaceutical pipeline. At a current estimated market share of 450 billion dollars annually, which is anticipated to exceed 1 trillion dollars by 2030, our protocol can become a key component in the biopharmaceutical pipeline. Disulfide mapping is also a critical attribute in detailed protein structure characterization. As currently most knowledge about disulfide bridges comes from much more complicated classical structural biology experiments like X-ray crystallography, complementary information provided by mass spectrometric analyses can be beneficial. Most proteins analyzed by the classical techniques are overexpressed recombinant and very often alkylated (alkylation improves the resolution for these imaging techniques) proteins that can result in errors of disulfide mapping due to scrambling or alkylation. MS can provide disulfide mapping for more complex and endogenous samples.

## Data Availability

Appropriate raw files and search results have been submitted to ProteomeXchange and can be accessed through accession: PXD046855. For potential review of the data, please use username: “reviewer_pxd046855@ebi.ac.uk” and password: “DhS5mphm”. The updated XlinkX node for Proteome Discoverer 3.1 will be made available through a service pack.

## Supplemental data

This article contains supplemental data.

## Conflict of interest

Y. H., Y. S., and R. V. are employees of Thermo Fisher Scientific, the manufacturer of the Orbitrap and the Proteome Discoverer platforms used in this work. The other authors declare no competing interests.

## Supplemental Data

Supplemental methods.docx
