# Supplementary material for: Fast and Accurate Disulfide Bridge Detection
Source: Mol Cell Proteomics. 2024 Apr 2;23(5):100759. doi: 10.1016/j.mcpro.2024.100759 (PMC11067345; doi:10.1016/j.mcpro.2024.100759)
Supplement: Supplemental methods.docx [file mmc1.docx]

**Supplementary Methods for “Fast and accurate disulfide bridge detection”**

Søren Heissel^1$^, Yi He^2^, Andris Jankevics^3*^, Yuqi Shi^2^, Henrik Molina^1^, Rosa Viner^2$^, Richard A. Scheltema^3$*^

1. Proteomics Resource Center, The Rockefeller University, 1230 York Avenue, New York, NY 10065, USA.
2. Thermo Fisher Scientific, 355 River Oaks Pkwy, San Jose, California 95134, United States.
3. Biomolecular Mass Spectrometry and Proteomics, Bijvoet Center for Biomolecular Research and Utrecht Institute for Pharmaceutical Sciences, University of Utrecht, Padualaan 8, 3584 CH Utrecht, The Netherlands.

* New address: Structural Proteomics Group, Department of Biochemistry and Systems Biology, University of Liverpool

**Introduction**

These supplemental methods describe the hydrolysis time evaluation experiment (Figure 1 C) and the modification landscape experiment (Supplemental Figure S2).

**Hydrolysis time evaluation**

LC-MS acquisition: Samples were separated by reverse phase-HPLC using a Thermo Scientific™ Easy nLC 1200 system connected to a pulled-emitter C18 column (0.075 mm x 120 mm, 3 µm particle size, 100 Å pore size (Nikkyo Technos)) at 300 nL/min flow rate. The hydrolyzed samples were analyzed on the Orbitrap Fusion Lumos mass spectrometer. Reverse phase separation was accomplished using a 70 min separation gradient of 2 – 35% solvent B (A: 0.1% FA; B: 80% ACN, 0.1% FA). Samples were analyzed using an EThcD-MS2 acquisition strategy. MS1 and MS2 scans were acquired in the Orbitrap with mass resolutions of 60,000. MS1 scan range was set to *m/z* 375 – 1700, standard AGC target, 50 ms maximum injection time and 60 s dynamic exclusion. MS2 scans in data dependent acquisition mode were set to an AGC target of 1e5, 118 ms max injection time, isolation window 1.6 *m/z*. Only precursors at charged states +2 to +7 were subjected to MS2.

Data analysis The hydrolysis time evaluation samples were searched in Proteome Discoverer v. 3.0 as described in the main text.

**Modification landscape analysis**

LC-MS acquisition: Samples were separated by reverse phase-HPLC using a Thermo Scientific™ Easy nLC 1200 system connected to a pulled-emitter C18 column (0.075 mm x 120 mm, 3 µm particle size, 100 Å pore size (Nikkyo Technos)) at 300 nL/min flow rate. The hydrolyzed samples were analyzed on the Orbitrap Fusion Lumos mass spectrometer. Reverse phase separation was accomplished using a 70 min separation gradient of 2 – 35% solvent B (A: 0.1% FA; B: 80% ACN, 0.1% FA). Samples were analyzed using stepped HCD-MS2 acquisition strategy using 15, 30, and 45 NCE. MS1 and MS2 scans were acquired in the Orbitrap with mass resolutions of 60,000. MS1 scan range was set to *m/z* 375 – 1750, standard AGC target, 50 ms maximum injection time and 60 s dynamic exclusion. MS2 scans in data dependent acquisition mode were set to a normalized AGC of 200%, 118 ms max injection time, isolation window 1.6 *m/z*. Only precursors at charged states +3 to +7 were subjected to MS2.

Data analysis: The spectra were queried against the trastuzumab sequence in an open search using MSFragger through Proteome Discoverer v. 2.5. Delta masses were binned and plotted against number of observations to study unknown modifications.
