# Supplementary material for: Fast and Accurate Disulfide Bridge Detection
Source: Mol Cell Proteomics. 2024 Apr 2;23(5):100759. doi: 10.1016/j.mcpro.2024.100759 (PMC11067345; doi:10.1016/j.mcpro.2024.100759)
Supplement: Supplementary_Review.pdf [file mmc2.pdf]

Supplementary data for “Fast and accurate disulfide bridge detection”

Søren Heissel[1$], Yi He[2], Andris Jankevics[3*], Yuqi Shi[2], Henrik Molina[1], Rosa Viner[2$], Richard A. Scheltema[3$*]

1. Proteomics Resource Center, The Rockefeller University, 1230 York Avenue, New York, NY 10065, USA.
2. Thermo Fisher Scientific, 355 River Oaks Pkwy, San Jose, California 95134, United States.
3. Biomolecular Mass Spectrometry and Proteomics, Bijvoet Center for Biomolecular Research and Utrecht Institute for Pharmaceutical Sciences, University of Utrecht, Padualaan 8, 3584 CH Utrecht, The Netherlands.

* New address: Structural Proteomics Group, Department of Biochemistry and Systems Biology, University of Liverpool

Contents

- **Supplementary Table 1 -** Used RAW files available in the PRIDE repository.
- **Supplementary Figure S1 –** Open search implementation
- **Supplementary Figure S2 –** Modifications associated with MAAH
- **Supplementary Figure S3 –** Further details on the effect of FAIMS integration in the data acquisition pipeline
- **Supplementary Figure S4** – Retention time alignment from peptide identifications
- **Supplementary Figure S5** – Crosslinker configuration for disulfide analysis in Proteome Discoverer

Supplementary Table 1: Used RAW files available in the PRIDE repository.

Figure	Raw file	Panels	Comments
1<br>Sx	Lysozyme_MAAH_NonReduced_5min.raw<br>Lysozyme_MAAH_NonReduced_7o5min.raw<br>Lysozyme_MAAH_NonReduced_10min.raw<br>Lysozyme_MAAH_NonReduced_15min.raw<br><br>**results_MAAH_psms.zip**	1B, 1C<br><br>Sx	Non-reduced Lysozyme recorded *without* FAIMS support.
2	trastuzumab_3_8_1ug_01.raw<br>trastuzumab_3_8FAIMS_1ug.raw<br>trastuzumab_3_8FAIMS_1ug_CV6075.raw<br><br>**results_FAIMS_csms_psms.zip**	C	Trastuzumab ran without and with FAIMS. Ultimately, only CV50/60 was used in all further experiments. “trastuzumab_3_8FAIMS_1ug” was recorded with FAIMS at CV 50/60 and is listed as “trastuzumab_3_8FAIMS_1ug_CV5060" in the zipped results.
4	Lysozyme_control.raw<br>Lysozyme_RT_1hr.raw<br>Lysozyme_RT_3hr.raw<br>Lysozyme_RT_6hr.raw<br>Lysozyme_37C_1hr.raw<br>Lysozyme_37C_3hr.raw<br>Lysozyme_37C_6hr.raw<br>Lysozyme_50C_1hr.raw<br>Lysozyme_50C_3hr.raw<br>Lysozyme_50C_6hr.raw<br><br>**results_scrambling_csms_psms.zip**	A,B	Scrambling experiment on Lysozyme.
5	trastuzumab_2_8FAIMS_1ug.raw<br>trastuzumab_3_8FAIMS_1ug.raw<br><br>**results_herceptin_csms_psms.zip**		Trastuzumab.
	Integrin_3_8FAIMS_1ug_1.raw<br><br>**results_integrin_csms_psms.zip**		Integrin
S1	20140201_EXQ00_RiSc_SA_STEVENHELA_01.raw<br><br>**20140201_EXQ00_RiSc_SA_STEVENHELA_01_mqevidence.txt**	A,B	Standard HeLa digest run on the Q Exactive HF. Published before in Scheltema *et al*, MCP,2014
S2	Trastuzumab_Stepped_HCD.raw<br><br>**Trastuzumab_Stepped_HCD_MSFragger_Search.xlsx**		Acid-hydrolyzed trastuzumab analyzed with stepped HCD fragmentation. Data was used for modification analysis and was subjected to open search using MSFragger.
Sx?	Lysozyme_0823_3_8_20min_1ug.raw<br>Lysozyme_0823_3_8_20min_1ug2.raw<br>Lysozyme_0823_3_8_30min_1ug2.raw<br>lysozyme0828_2_8FAIMS_1hr1ug_20230828143855.raw<br>lysozyme0828_3_8FAIMS_1hr1ug2.raw<br><br>**results_shortgradient_csms_psms.zip**		Reduced gradient lengths. Not used in figures, discussed in the main text.

Supplementary Figures

Heissel et al; Supplementary Figure S1

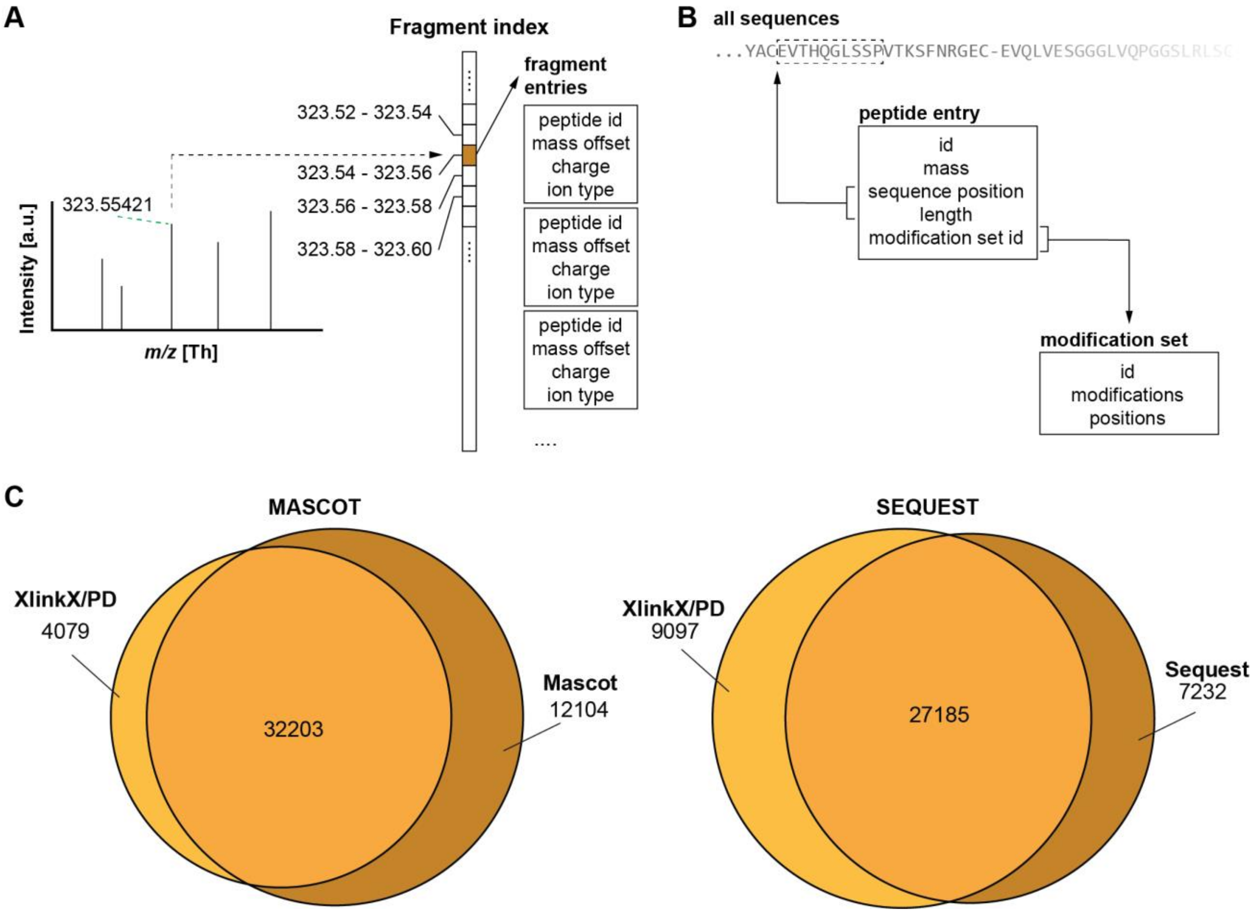

Supplementary Figure S1 – Open search implementation. (A) The fragment index implementation initially digests all protein sequences and for each peptide generates the theoretical fragments that are stored in a list sorted on fragment mass. For each peak in the fragmentation spectrum, associated peptides can almost instantaneously be recalled. In our implementation each peptide entry is restricted to 16 bytes, meaning that for 5 million peptides only 80 Mb is used. (**B)** For each peptide meta data is maintained that allows for construction of the sequence and the modification profile. Additionally, an extra list is maintained sorted on mass that allows for a classical approach for searching based on the precursor mass. **C.** When using the search engine in linear peptide search mode allows for comparison to existing search engines.

Examination of the overlap of the identified peptides shows good overlap between XlinkX/PD and MASCOT or SEQUEST. In cases where the other search engines identify extra peptides in close to all cases our open search detects the same peptide identity but removes them due to quality concerns.

Heissel et al; Supplementary Figure S2

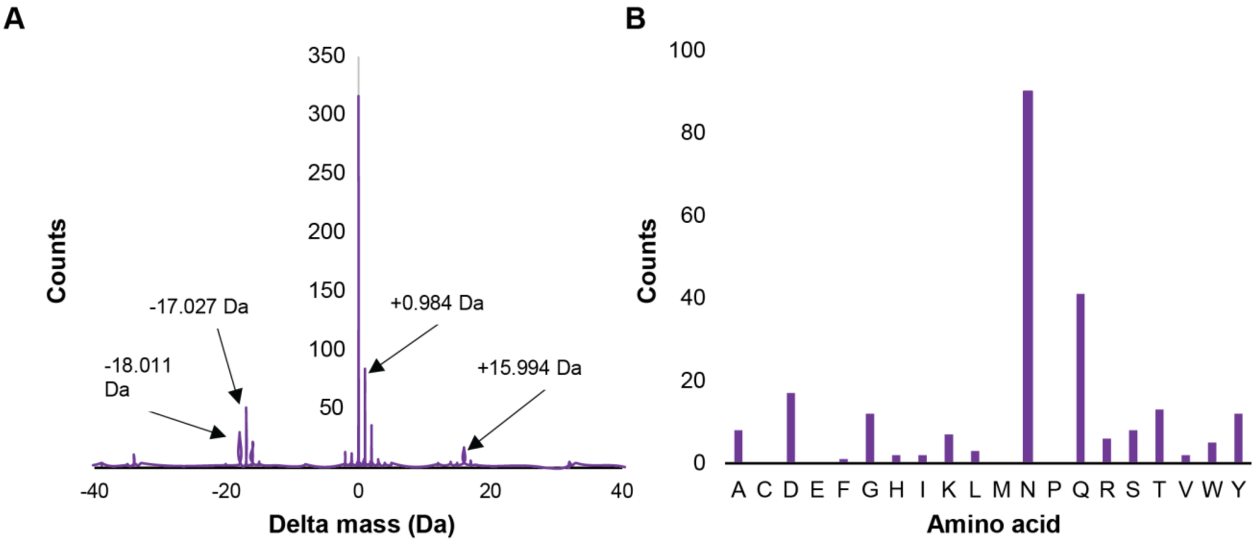

Supplementary Figure S2 – Modifications induced by MAAH. (A) Mass shifts binned in 0.01 Da intervals plotted against the number of observations. The results were generated from MAAH-hydrolyzed Trastuzumab fragmented with HCD and searched by MSFragger in open search mode. **(B)** Number of -17 Da loss for each amino acid based on PSMs. Data is produced from MAAH-hydrolyzed Trastuzumab fragmented with EThcD and searched by SEQUEST HT with loss of ammonia (-17.027 Da) as a variable modification.

Heissel et al; Supplementary Figure S3

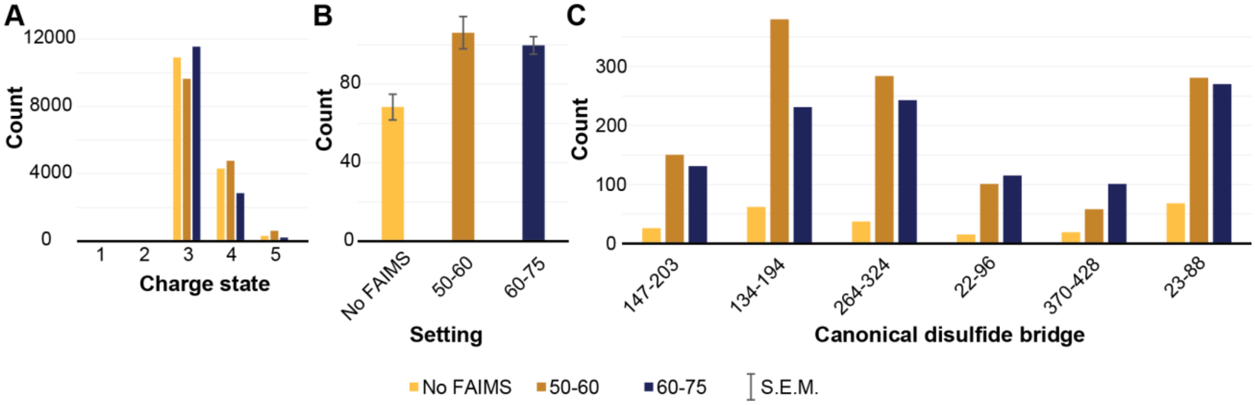

Supplementary Figure S3 – Further details on the effect of FAIMS integration in the data acquisition pipeline. (A) Comparing the for-fragmentation-selected precursor charge states shows no major effect in the selection of the precursor (note, the charge state is determined during data acquisition). **(B)** The scores assigned to the identifications significantly increases with FAIMS compared to NO FAIMS. This is linked to the increase in number of identified precursors as detailed in Figure 2C. **(C)** The number of CSMs significantly increases with FAIMS on per unique disulfide bridge in Trastuzumab (denoted on the x-axis as the protein positions).

Heissel et al; Supplementary Figure S4

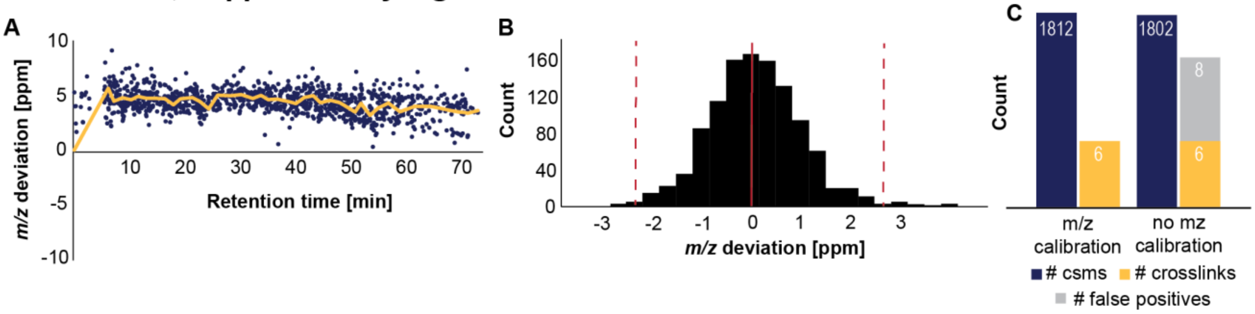

Supplementary Figure S4 – Retention time alignment from peptide identifications. A. Dot plot of m/z deviation over the entire MS run. From the point cloud spline can be fit that describes the average *m/z* drift over the retention time. The spline is used to correct the precursor masses of all fragmentation spectra prior to the crosslinked peptide identification step. **B.** The distribution of precusor masses was polted after recalibration in ppm, the full population adheres to a normal distrubution from which the edges can be estimated in a data driven fashion. We utilize interquartile range fences, where the interquartile range multiplied by a factor of 3 is added (right hand fence) and subtracted (left hand fence) to quartile3. **(C)** Number of CSMs, crosslinks, and false positives were compared with or without m/z calibration.

Heissel et al; Supplementary Figure S5

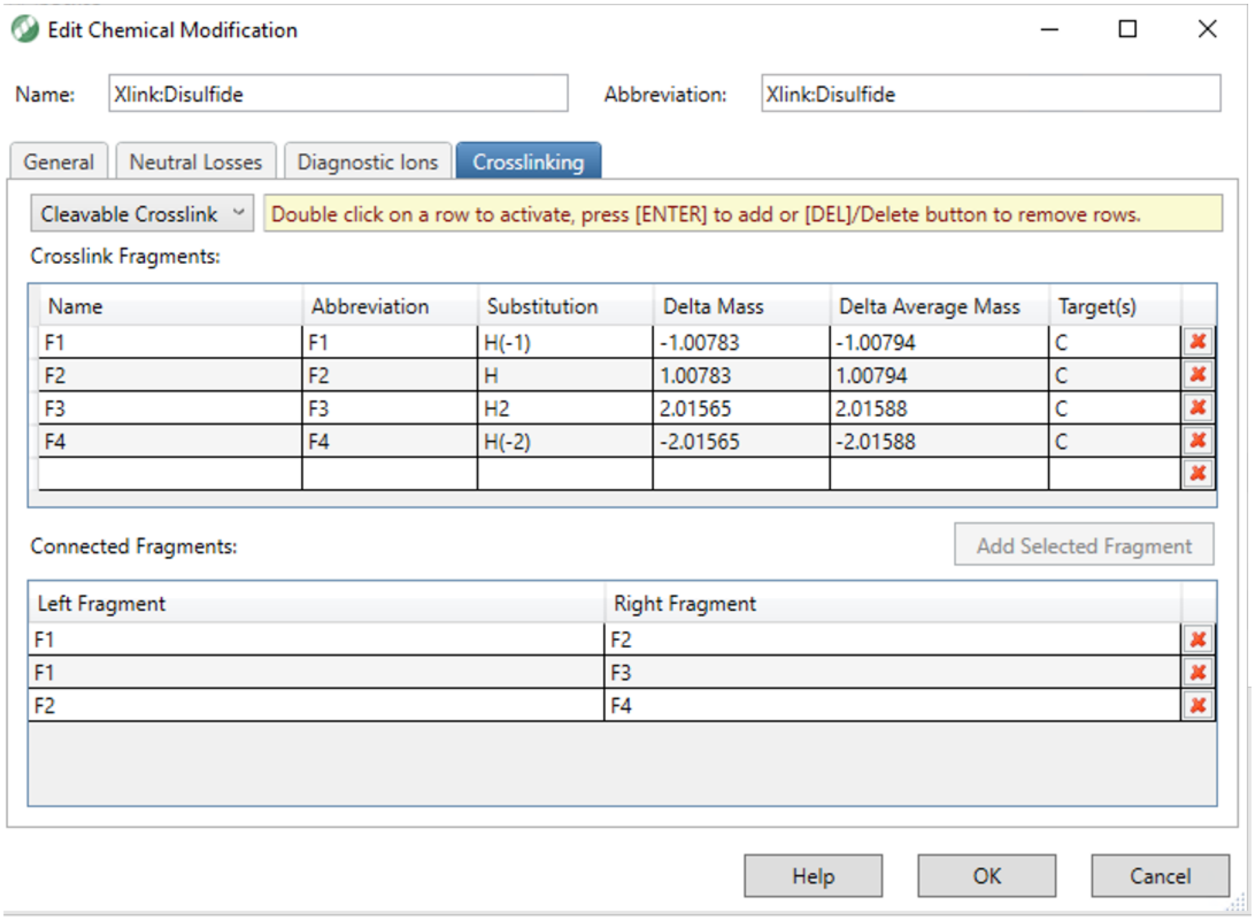

Supplementary Figure S5 – Crosslink Fragment configuration in Proteome Discoverer. Overview of how the crosslink fragments should be configured in Proteome Discoverer for disulfide bond analysis using XlinkX.
